# In silico structural and functional characterization of hypothetical proteins from Monkeypox virus

**DOI:** 10.1186/s43141-023-00505-w

**Published:** 2023-04-26

**Authors:** Kajal Gupta

**Affiliations:** Department of Biochemistry, Daulat Ram College, University of Delhi, Delhi, India

**Keywords:** Monkeypox, Monkeypox virus, Hypothetical proteins, Bioinformatics analysis, Drug target identification

## Abstract

**Background:**

Monkeypox virus is a small, double-stranded DNA virus that causes a zoonotic disease called Monkeypox. The disease has spread from Central and West Africa to Europe and North America and created havoc in some countries all around the world. The complete genome of the Monkeypox virus Zaire-96-I-16 has been sequenced. The viral strain contains 191 protein-coding genes with 30 hypothetical proteins whose structure and function are still unknown. Hence, it is imperative to functionally and structurally annotate the hypothetical proteins to get a clear understanding of novel drug and vaccine targets. The purpose of the study was to characterize the 30 hypothetical proteins through the determination of physicochemical properties, subcellular characterization, function prediction, functional domain prediction, structure prediction, structure validation, structural analysis, and ligand binding sites using Bioinformatics tools.

**Results:**

The structural and functional analysis of 30 hypothetical proteins was carried out in this research. Out of these, 3 hypothetical functions (Q8V547, Q8V4S4, Q8V4Q4) could be assigned a structure and function confidently. Q8V547 protein in Monkeypox virus Zaire-96-I-16 is predicted as an apoptosis regulator which promotes viral replication in the infected host cell. Q8V4S4 is predicted as a nuclease responsible for viral evasion in the host. The function of Q8V4Q4 is to prevent host NF-kappa-B activation in response to pro-inflammatory cytokines like TNF alpha or interleukin 1 beta.

**Conclusions:**

Out of the 30 hypothetical proteins of Monkeypox virus Zaire-96-I-16, 3 were annotated using various bioinformatics tools. These proteins function as apoptosis regulators, nuclease, and inhibitors of NF-Kappa-B activator. The functional and structural annotation of the proteins can be used to perform a docking with potential leads to discover novel drugs and vaccines against the Monkeypox. In vivo research can be carried out to identify the complete potential of the annotated proteins.

**Supplementary Information:**

The online version contains supplementary material available at 10.1186/s43141-023-00505-w.

## Background

**Fig. 1:**
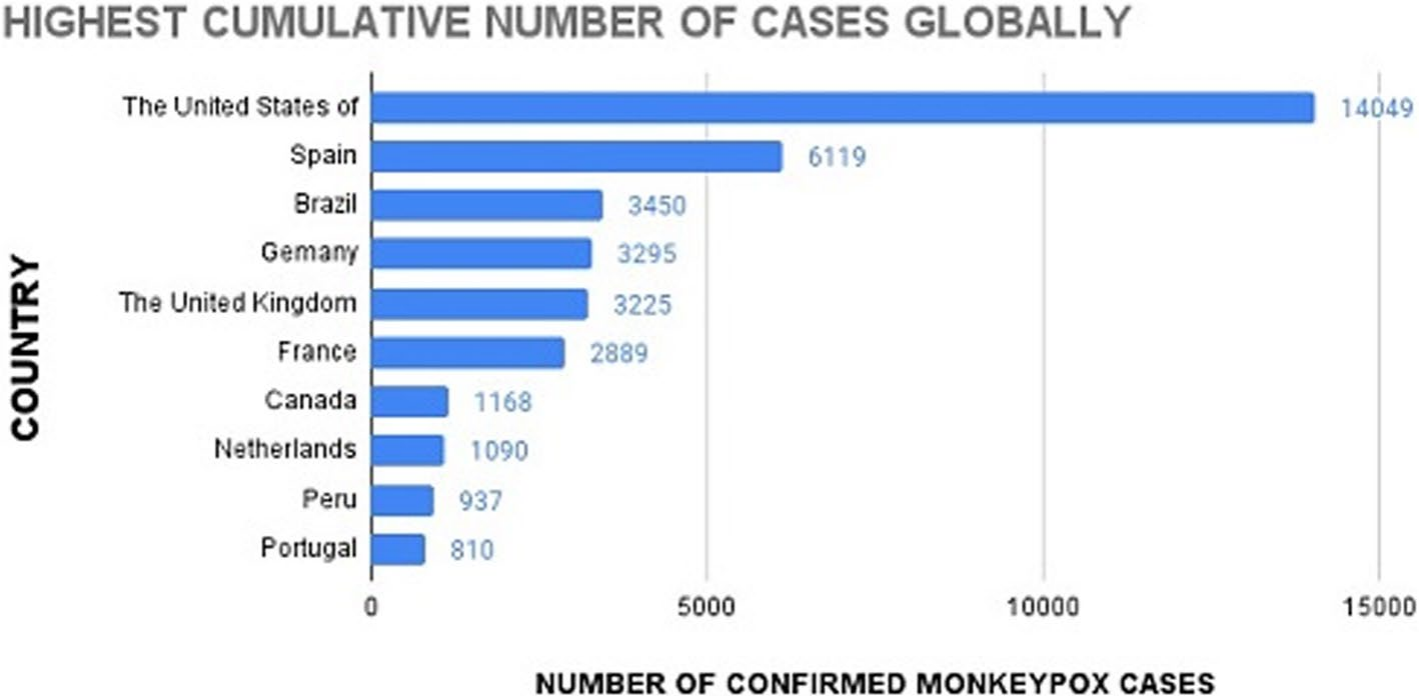
Ten countries with highest cumulative number of Monkeypox cases globally (World Health Organization)

Monkeypox (MPX) is a viral disease caused by the Monkeypox virus (MPXV), which can spread from vertebrates to humans and vice versa. It was earlier prevalent in Central and West Africa. Due to its similarity with symptoms in smallpox, MPX was also known as “Monkey smallpox.” The major symptoms of MPX are high fever, headache, lymphadenopathy, and systemic blisters and pustules [1]. The mortality rate of Monkeypox is 1–10% [2, 3]. Since May 2022, Monkeypox cases have been found not only in the countries where the disease was endemic but also in certain countries of Europe and North America. From 1 January 2022 to 22 August 2022, 41,664 laboratory-confirmed cases and 12 deaths were reported in the world, according to the Multi-country outbreak of Monkeypox report published by the World Health Organization (WHO). Ten countries including the USA, Spain, Brazil, and others account for 88.9% of the cases reported globally (Fig. 1) [4].

The increased emergence of the disease has created havoc among a wider audience leading to higher demand for potential drugs and vaccines. There are currently no FDA-approved drugs for Monkeypox. The FDA-approved drugs for smallpox tecovirimat and brincidofovir are administered to patients with Monkeypox. However, low viral resistance barrier can render the drug useless when used indiscriminately. An FDA-approved vaccine JYNNEOS vaccine is being marketed for prevention of Monkeypox in individuals older than 18 years and susceptible to the infection [5]. Thus, it has become essential to study the genome of the Monkeypox virus in-depth for understanding new potential drug and vaccine targets in MPXV which can be administered safely to individuals of all age groups. In this research paper, we studied the hypothetical proteins in MPXV to discover new drug and vaccine targets in MPXV.

Monkeypox virus (MPXV) is a poxvirus belonging to the *Orthopoxvirus* genus. The 200 × 250 nm sized virus particle has an oval or brick shape [6] and produces two infectious particles during replication: intracellular mature viral particle and extracellular enveloped viral particle. The structure of an intracellular mature viral particle comprises a lipoprotein envelope around the viral core and a lateral body rich in proteins. It is quite stable in the external environment and is released in the environment by cell lysis. It usually aids the virus in disease transmission between different animals. On the other hand, an extracellular enveloped viral particle comprises a lipid membrane wrapped around the intracellular mature viral particle which is formed from the transport Golgi apparatus or endosomes [7].

The replication cycle of poxvirus is completed in the cytoplasm. The host cell invasion takes place in 3 steps: adsorption, membrane fusion, and core invasion. Although specific cell receptors are not known for MPXV invasion, it has been identified that in the case of vaccinia virus (VACV), which is similar to MPXV, the adsorption on the cell surface occurs by four viral proteins namely D8, A27, A26, and H3. D8 binds to chondroitin [8], A26 binds to laminin [9], and A26 and H3 bind to heparan [10] mediating the adsorption of the virus on the host cell surface, which is followed by membrane fusion and core invasion.

The viral proteins A16, A21, A28, F9, G3, G9, H2, J5, L1, L5, and O3 form an entry fusion complex in VACV to allow the invasion of intracellular mature viral particle and Extracellular enveloped viral particle in the host cell. Except for O3, all the other viral proteins also play an important role in poxvirus replication [11, 12]. Following the membrane invasion, the viral core enters the cytoplasm and is de-hulled by the action of certain viral proteins like A16L, A21L, A28L, F9L, G3L, G9R, H2R, J5L, and L5R, to initiate viral biosynthesis [12, 13].

Monkeypox virus Zaire-96-I-16(MPXV-ZAI) [NCBI:txid619591] has a non-segmented, linear, double-stranded DNA genome consisting of 196.858 kilobase pairs (Kbp) [NCBI Reference Sequence: NC_003310.1]. The genome comprises a total of 191 genes and 175 protein clusters [14]. There are 190 non-overlapping ORFs of ≥ 60 amino acids, and the GC content is approximately 31.1%. The coding region of MPXV-ZAI is around 195,118 bp long. *Homo sapiens* are the known hosts for MPXV [15]. One hundred ninety-one viral segments of MPXV-ZAI can be classified into various categories according to their function. Thirty proteins out of these unknown viral segments are hypothetical proteins, which have been studied in this research. The protein length, Uniprot Id, and locus tag are listed in Table S1 [16].

## Methods

### Sequence retrieval

We analyzed the genome of the Monkeypox virus Zaire-96-I-16(MPXV-ZAI)[NCBI:txid619591] and found 161 protein-coding genes (http://www.ncbi.nlm.nih.gov/genome) and a total of 30 proteins as hypothetical proteins (HPs). The FASTA sequences of all 30 HPs proteins were retrieved from Uniprot (http://www.uniprot.org) [17].

### Physicochemical properties of HPs

The physicochemical parameters of all 30 HPs were studied using Expasy’s ProtParam server [18] (https://web.expasy.org/protparam/), which were then used for theoretical measurements of various parameters such as molecular weight, theoretical isoelectric point, extinction coefficient [19], instability index [20], aliphatic index, and grand average of hydropathicity (GRAVY) [21]. The extinction coefficient measures the amount of light that proteins absorb at a certain wavelength. Theoretical estimation of the stability of a protein in a test tube can be known by the instability index. The relative volume occupied by aliphatic side chain amino acids in the protein can be known by the aliphatic index of a protein. The GRAVY score for a peptide or protein is calculated as the sum of the hydropathy values of all of the amino acids, divided by the number of residues in the protein sequence. The predicted properties of HPs are listed in Table S2.

### Subcellular localization and transmembrane helices prediction

The subcellular location of a protein usually determines the function of proteins. Thus, the prediction of subcellular localization from protein sequence can be useful to determine the protein function which can further aid in developing antiviral drugs and vaccines. The protein can be present in the outer membrane, inner membrane, capsid, periplasm, cytoplasm, or extracellular space [22]. The subcellular localization of HPs were predicted using Virus-PLoc [23–26], TMHMM [27–29], and HMMTOP [30, 31].

Virus-PLoc (http://www.csbio.sjtu.edu.cn/bioinf/virus/) predicts the subcellular location of viral proteins within a host and virus-infected cells. The predictor classifies the viral protein locations in the following categories: (1) cytoplasm, (2) endoplasmic reticulum, (3) extracellular, (4) inner capsid, (5) nucleus, (6) outer capsid, and (7) plasma membrane. The prediction approach is by fusing PseAA (pseudo amino acid) composition.

TMHMM (https://services.healthtech.dtu.dk/service.php?TMHMM-2.0) predicts the transmembrane helices in proteins. The TMHMM prediction tool gives information about the most probable location and orientation of transmembrane helices in the protein sequence. It works on the algorithm called N-best that sums over all paths through the model with the same location and direction of the helices.

HMMTOP (http://www.enzim.hu/hmmtop/index.php) predicts the localization of helical transmembrane segments and the topology of helical transmembrane proteins. The predicted subcellular localization of HPs is listed in Table S3.

### Function prediction using sequence analogy

The most basic step to understanding the function of an unknown protein is by looking for its structural homologs in different genomics and proteomics-based databases. This approach works on the hypothesis that an unknown protein with sequence analogy to a known protein may have a similar function as the known protein. The sequence similarity search was performed via protein BLAST (pBLAST) against the non-redundant database. Generally, HPs contain low identity as compared to other known or annotated proteins [32, 33]. The predicted functions according to sequence analogy through pBLAST are listed in Table S4.

### Function and functional domain prediction through sequence analysis

Precise function of a protein can be determined using the information about functional domain in the HPs, various bioinformatics tools like SMART [34, 35], MOTIF-SCAN [36], INTERPROSCAN [37–39], pfp-fundseqe [25, 40, 41], PFAM [42–46], CATH [47, 48], and SUPERFAMILY [49, 50] were used to detect functional domains in HPs and classify the 30 HPs into family and superfamily.

SMART (a Simple Modular Architecture Research Tool) identifies and annotates the genetically mobile domains and analyses domain architectures.

Motif-Scan (including HAMAP profiles, Prosite patterns, Pfam HMMs (local models), and Pfam HMMs (global models)) were used to find the motifs in a protein sequence.

InterProScan uses protein sequence in FASTA format to predict the family to which the unknown protein belongs and the domain present in it. The predicted functional domains in HPs are listed in Table S5.

### Structure prediction and validation

The structure of 30 HPs were predicted using Phyre2 Batch processing [51]. Phyre2 utilizes an extensive remote homology detection method to build 3D models of unknown proteins. Seven protein models were confidently predicted by Phyre2. The models were validated using Procheck [52–55] and SWISS-Model QMEANDisCo scoring function [56].The QMEANDisCo scoring function of 7 predicted models is listed in Table S6.

### Function annotation using structural analysis

Protein function predicted from structure gives more valuable information than protein function predicted through sequence analysis [57]. The structural analysis of 7 predicted protein models was done using DeepFRI [58] and COACH [59, 60].

The structure-based molecular function and biological process predicted using DeepFRI are listed in Table S7.

The ligand and consensus binding residues of predicted models are listed in Table S8.

## Results

Monkeypox virus Zaire-96-I-16 (MPXV-ZAI) comprises 191 protein-coding genes. Out of these, 30 proteins are regarded as hypothetical proteins. In this study, these proteins were studied using various bioinformatics tools for annotating their function in viral infection. The amino acid length of 30 hypothetical proteins ranges from 64 for shortest protein to 737 for longest protein. Expasy’s ProtParam tool was used to estimate the physicochemical properties of the HPs. The subcellular localization and presence of transmembrane helices revealed the presence of most of the HPs in the inner layer of the host cell and a few in the Endoplasmic reticulum and host cytoplasm. Approximately half of the HPs were predicted as transmembrane proteins with 1–2 transmembrane helices. The function of 5 hypothetical proteins could be predicted with confidence (≥ 4 software predictions). The function of these proteins and their subcellular localization are listed in Table 1.

**Table 1:** Hypothetical proteins with confidently predicted function (≥ 4 software prediction) and subcellular localization

S.NO	Protein name	UniprotKB identifier	Function	Virus-PLoc prediction
1	Hypothetical protein MPXVgp033	Q8V547	Apoptosis regulators	Endoplasmic Reticulum
2	Hypothetical protein MPXVgp037	Q8V543	Telomere binding protein	Endoplasmic Reticulum
3	Hypothetical protein MPXVgp043	Q8V537	Cell-to-cell spread of the virus	Inner layer
4	Hypothetical protein MPXVgp081	Q8V501	Early virion morphogenesis and formation or elongation of crescent membranes	Plasma membrane
5	Hypothetical protein MPXVgp165	Q8V4S4	Nuclease	Plasma membrane

The function of additional 8 proteins could be predicted with uncertainty (= 3 software predictions). The function of these proteins and their subcellular localization are listed in Table 2.

**Table 2:** Hypothetical proteins with confidently predicted function (= 3 software prediction) and subcellular localization

S.NO	Protein name	UniprotKB identifier	Function	Virus-PLoc prediction
1	Hypothetical protein MPXVgp016	Q8V563	Inhibitor of host NF-kappa-B activation	Endoplasmic reticulum
2	Hypothetical protein MPXVgp026	Q8V554	Immunosuppressive activities	Endoplasmic reticulum
3	Hypothetical protein MPXVgp056	Q8V524	Virion assembly	Cytoplasm
4	Hypothetical protein MPXVgp066	Q8V514	Membrane protein	Endoplasmic reticulum
5	Hypothetical protein MPXVgp067	Q8V513	Binds to the hairpin form of the viral telomeric sequence	Plasma membrane
6	Hypothetical protein MPXVgp074	Q8V506	Nuclease	Inner layer
7	Hypothetical protein MPXVgp147	Q8V4U2	Virulence	Outer capsid
8	Hypothetical protein MPXVgp185	Q8V4Q4	Prevent host NF-kappa-B activation	Cytoplasm

The structure prediction and structure analysis of 30 hypothetical proteins were done. Out of 30 proteins, the structure of 7 proteins were confidently predicted (> 65% of residues modeled with confidence > 90%). The structure of these 7 proteins were validated and analyzed through various bioinformatics software.

## Discussion

In this study, we carried out the structural and functional annotation of 30 hypothetical proteins from the Monkeypox virus Zaire-96-I-16(MPXV-ZAI). Physicochemical properties predicted through Expasy’s ProtParam server revealed that the isoelectric point of the HPs ranges from 3.48 to 9.49. The isoelectric point is the pH at which the net charge on a protein molecule is zero. Protein at its isoelectric point is mildly insoluble, compact, and stable, which leads to protein getting crystallized [61].

The extinction coefficient of protein ranges from 1490 M-1 Cm-1 to 80,540 M-1 Cm-1 at 280 nm. Analysis of the extinction coefficient of a protein helps to determine the protein–protein interaction and protein–ligand interaction in solution [19]. The instability index provides information about the stability of a protein in the test tube. A value of greater than 40 indicates that a protein is unstable and a value of less than 40 indicates a stable protein. Out of 40 hypothetical proteins, 13 proteins were regarded as unstable and 17 proteins are stable in the test tube. The relative volume occupied by the aliphatic side chains in a protein is indicated by the Aliphatic index. It can be used to determine the thermostability of globular proteins [62].The aliphatic index of HPs ranges from 36.56 to 177.83. GRAVY (Grand Average of Hydropathy) of the HPs ranges from − 1.602 to 1.223.

The detailed analysis of 3 of the HPs from the Monkeypox virus whose function and structure (through Phyre2) could be annotated with confidence is mentioned as follows.

### Q8V547

Q8V547 is predicted as an apoptosis regulator that promotes viral replication in the host cell and has an immunoglobulin-like domain. Also, it may act as an inhibitor of NLR-mediated interleukin-1 beta/IL1B production in infected cells. The function is confidently predicted by 5 software. The Virus Ploc software detected its subcellular location in the endoplasmic reticulum of the virus-infected cell with no transmembrane helices. The predicted 3-dimensional structure showed that 144 residues (66% of the sequence) have been modeled with 100.0% confidence by the single highest scoring template (Figure S1), and validation through the Ramachandran plot revealed 96.50% residues in the most favored regions and 2.8% residues in additional allowed regions (Figure S2). There are no residues in the outlier region. The QMEANDisCo Global Score of the predicted model was 0.68 ± 0.07(above the cutoff range of 0.50). The structure analysis through COACH predicted the ligand for this protein to be a peptide. The Gene Ontology structure-based analysis predicted the biological process of Q8V547 in cellular metabolism and primary metabolism.

### Q8V4S4

Q8V4S4 is predicted as a nuclease responsible for viral evasion. A DNA-binding domain in the protein has also been detected in the functional domain annotation. The function has been annotated confidently by 4 function prediction software. A confidently predicted 3-dimensional structure of the protein with 97% of residues modeled at > 90% confidence is selected (Figure S3). Validation of the structure through the Ramachandran plot revealed 73% in the most favored region, 18.6% residues in the additional allowed region, and 4% residues in the outlier region (Figure S4). The Swiss model QMEANDisCo Global Score of the predicted structure was 0.59 ± 0.05 (above the cutoff range of 0.50). The analysis of the predicted structure through COACH revealed GERAN-8-YL GERAN as the ligand for the protein. The Gene Ontology structure-based analysis of Q8V4S4 predicted the molecular function as organic cyclic and heterocyclic compound binding and a biological role in organic substance metabolism, primary metabolism, nitrogen compound metabolism, macromolecule metabolism, cellular metabolism, and cellular macromolecule metabolism.

### Q8V4Q4

The function of Q8V4Q4 as predicted confidently by 3 software is to prevent host NF-kappa-B activation in response to pro-inflammatory cytokines like TNF alpha or interleukin 1 beta. A 4 helical cytokine binding motif is predicted by pfp-fundseqe. A 3-dimensional model with 133 residues (87% of the sequence) has been modeled with 100.0% confidence by the single highest scoring template (Figure S5). The model as validated by Ramachandran plot revealed 84.1% residues in most favored regions, 12.1% in additional allowed regions, and 1.5% in outlier region (Figure S6). QMEANDisCo Global Score of the predicted model is 0.63 ± 0.07(above the cutoff range of 0.50). The structural analysis of the model on COACH revealed 3-[3-(4-chloro-3,5-dimethylphenoxy)PROPYL]-1-benzothiophene-2-carboxylic acid as the ligand of the protein. The structure analysis of the 3D model revealed protein binding as the molecular function of the protein and regulation of cellular process as the biological process.

## Conclusion

This research worked on predicting the structure and function of hypothetical proteins of Monkeypox virus Zaire-96-I-16 using bioinformatics tools and software. Three of the 30 hypothetical proteins (Q8V547, Q8V4S4, Q8V4Q4) were confidently annotated. Q8V547 protein was predicted as an apoptosis regulator which promotes viral replication in the infected host cell.

Q8V4S4 was predicted as a nuclease responsible for viral evasion in the host. The function of Q8V4Q4 was to prevent host NF-kappa-B activation in response to pro-inflammatory cytokines like TNF alpha or interleukin 1 beta. Further studies are required to unravel the complete potential of these proteins as potential drug targets and vaccines. Since these proteins were predicted to be involved in protection and proliferation of the virus inside a host, they can prove to be good targets for drugs and vaccines for protection against the disease.

## Supplementary Information

**Additional file 1: Supplementary Figure 1 (Figure S1).** 3D structure of hypothetical protein Q8V547 predicted from Phyre2. **Supplementary Figure 2 (Figure S2): **Evaluation of 3D structure of Hypothetical Protein Q8V547 through Ramachandran plot. **Supplementary Figure 3 (Figure S3): **3D structure of hypothetical protein Q8V4S4 predicted from Phyre2. **Supplementary Figure 4 (Figure S4): **Evaluation of 3D structure of Hypothetical Protein Q8V4S4 through Ramachandran plot. **Supplementary Figure 5 (Figure S5): **3D structure of hypothetical protein Q8V4Q4 predicted from Phyre2. **Supplementary Figure 6 (Figure S6): **Evaluation of 3D structure of Hypothetical Protein Q8V4Q4 through Ramachandran plot.

**Additional file 2: Supplementary Table 1 (Table S1).** The table presents the 30 Hypothetical proteins of Monkeypox virus Zaire-96-I-16 along with the Uniprot Id, Locus tag and protein length. **Supplementary Table 2 (Table S2):** The table reports the physiochemical properties of 30 hypothetical proteins as predicted by Expasy’s Protparam server. **Supplementary Table 3 (Table S3):** The table details the list of predicted subcellular location and presence of transmembrane helices in 30 Hypothetical proteins. **Supplementary Table 4 (Table S4):** The table reports the function of 30 hypothetical proteins as predicted by Protein BLAST. **Supplementary Table 5(Table S5):** The table presents the function and functional domain of 30 Hypothetical proteins as annotated by SMART,Motif Scan, pfp- fundseqe, InterProscan, PFAM, CATH and Superfamily. **Supplementary Table 6 (Table S6): **The table entails the QMEANDisCo Global Score of the predicted 3 dimensional structure of 7 Hypothetical proteins. **Supplementary Table 7 (Table S7):** The table reports the Structure-Based Molecular Function and Structure-Based Biological Process of the 7 Hypothetical proteins from there predicted 3 dimensional structure. **Supplementary Table 8 (Table S8): **The table presents the ligand binding site prediction of 7 Hypothetical proteins of Monkeypox virus.

## Data Availability

All data generated or analyzed during this study are included in this published article [and its supplementary information files].

## Abbreviations

MPX: Monkeypox
MPXV: Monkeypox virus
VACV: Vaccinia virus
MPXV-ZAI: Monkeypox virus Zaire-96-I-16
HPs: Hypothetical proteins
GRAVY: Grand average of hydropathicity
