# Correction: In silico structural and functional characterization of hypothetical proteins from Monkeypox virus

**DOI:** 10.1186/s43141-023-00512-x

**Published:** 2023-05-05

**Authors:** Kajal Gupta

**Affiliations:** Department of Biochemistry, Daulat Ram College, University of Delhi, Delhi, India

**Correction: J Genet Eng Biotechnol 21, 46 (2023)**

**https://doi.org/10.1186/s43141-023-00505-w**

In this article [1], the title was incorrectly given as “Functional characterization of hypothetical proteins from Monkeypox virus” but should have been “In silico structural and functional characterization of hypothetical proteins from Monkeypox virus”.

The original article has been corrected.
