# Supplementary material for: In silico structural and functional characterization of hypothetical proteins from Monkeypox virus
Source: J Genet Eng Biotechnol. 2023 Apr 26;21:46. doi: 10.1186/s43141-023-00505-w (PMC10133424; doi:10.1186/s43141-023-00505-w)
Supplement: Supplementary file 1 — Additional file 1: Supplementary Figure 1 (Figure S1). 3D structure of hypothetical protein Q8V547 predicted from Phyre2. Supplementary Figure 2 (Figure S2): Evaluation of 3D structure of Hypothetical Protein Q8V547 through Ramachandran plot. Supplementary Figure 3 (Figure S3): 3D structure of hypothetical protein Q8V4S4 predicted from Phyre2. Supplementary Figure 4 (Figure S4): Evaluation of 3D structure of Hypothetical Protein Q8V4S4 through Ramachandran plot. Supplementary Figure 5 (Figure S5): 3D structure of hypothetical protein Q8V4Q4 predicted from Phyre2. Supplementary Figure 6 (Figure S6): Evaluation of 3D structure of Hypothetical Protein Q8V4Q4 through Ramachandran plot. [file 43141_2023_505_MOESM1_ESM.pdf]

Journal name - Journal of Genetic Engineering and Biotechnology

Manuscript Title - IN-SILICO STRUCTURAL AND FUNCTIONAL CHARACTERIZATION OF HYPOTHETICAL PROTEINS FROM MONKEYPOX VIRUS

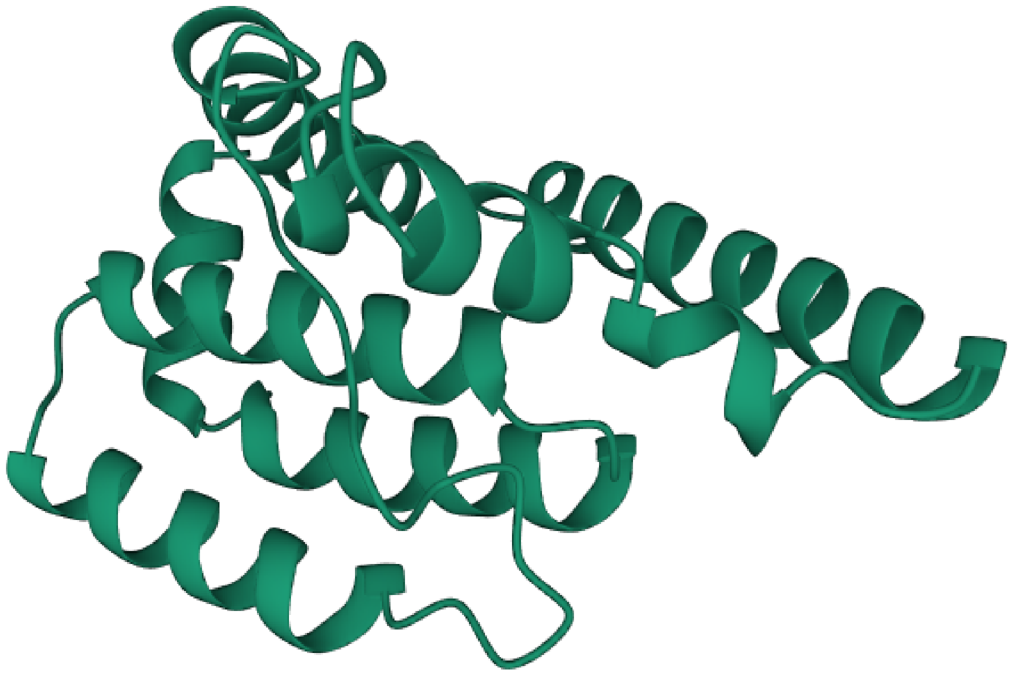

Supplementary Figure 1 (Figure S1) : 3D structure of hypothetical protein Q8V547 predicted from Phyre2.

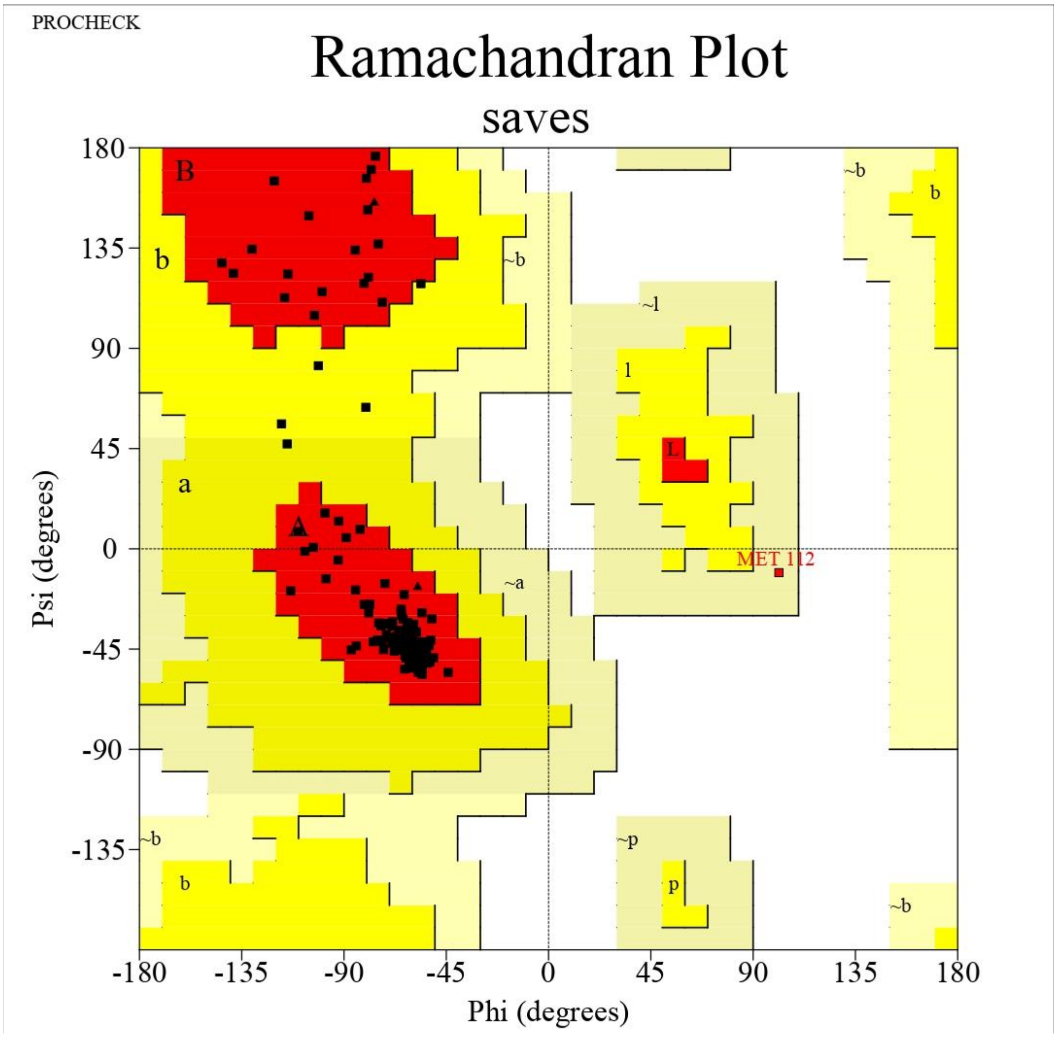

Supplementary Figure 2 (Figure S2) : Evaluation of 3D structure of Hypothetical Protein Q8V547 through Ramachandran Plot.

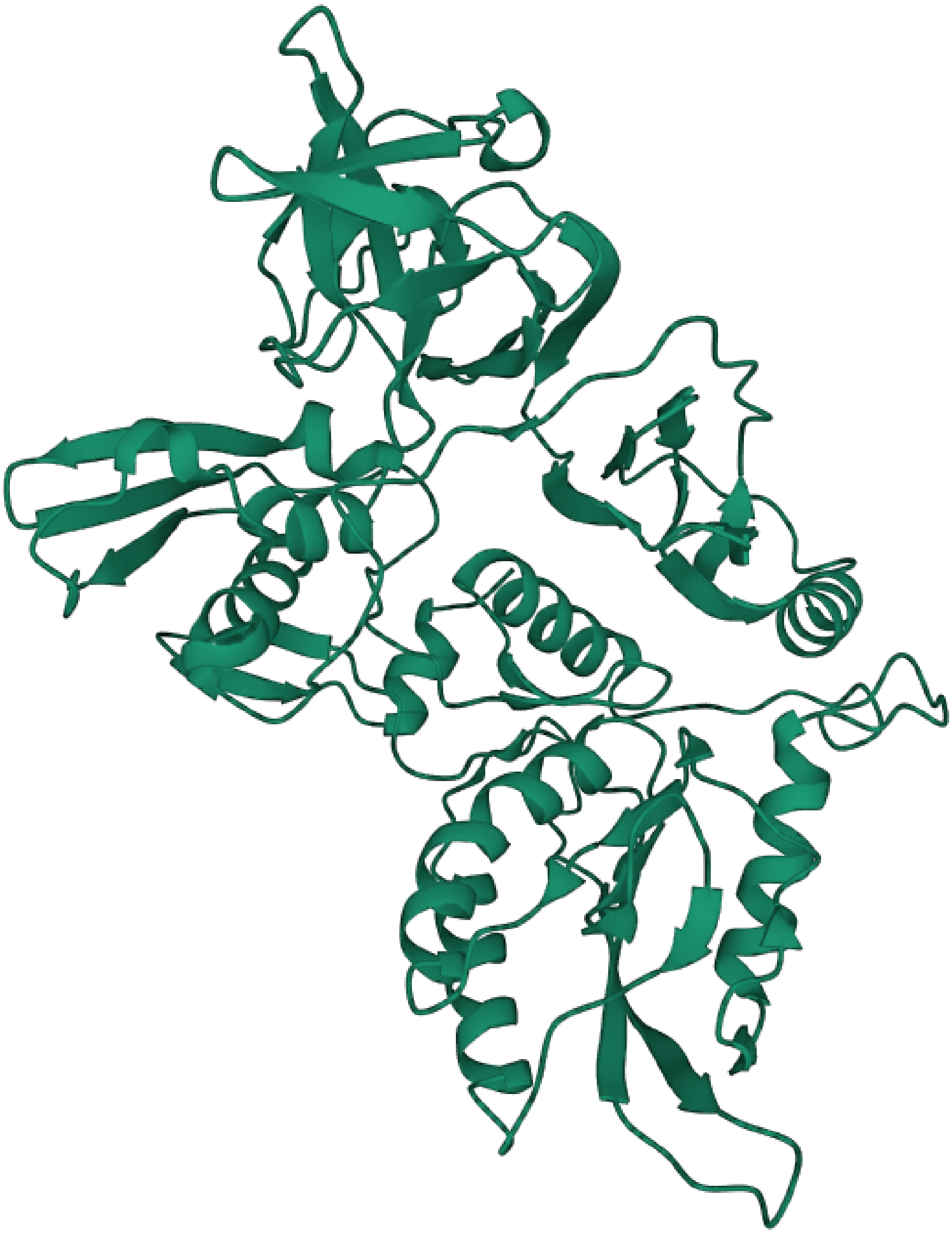

Supplementary Figure 3 (Figure S3) : 3D structure of hypothetical protein Q8V4S4 predicted from Phyre2.

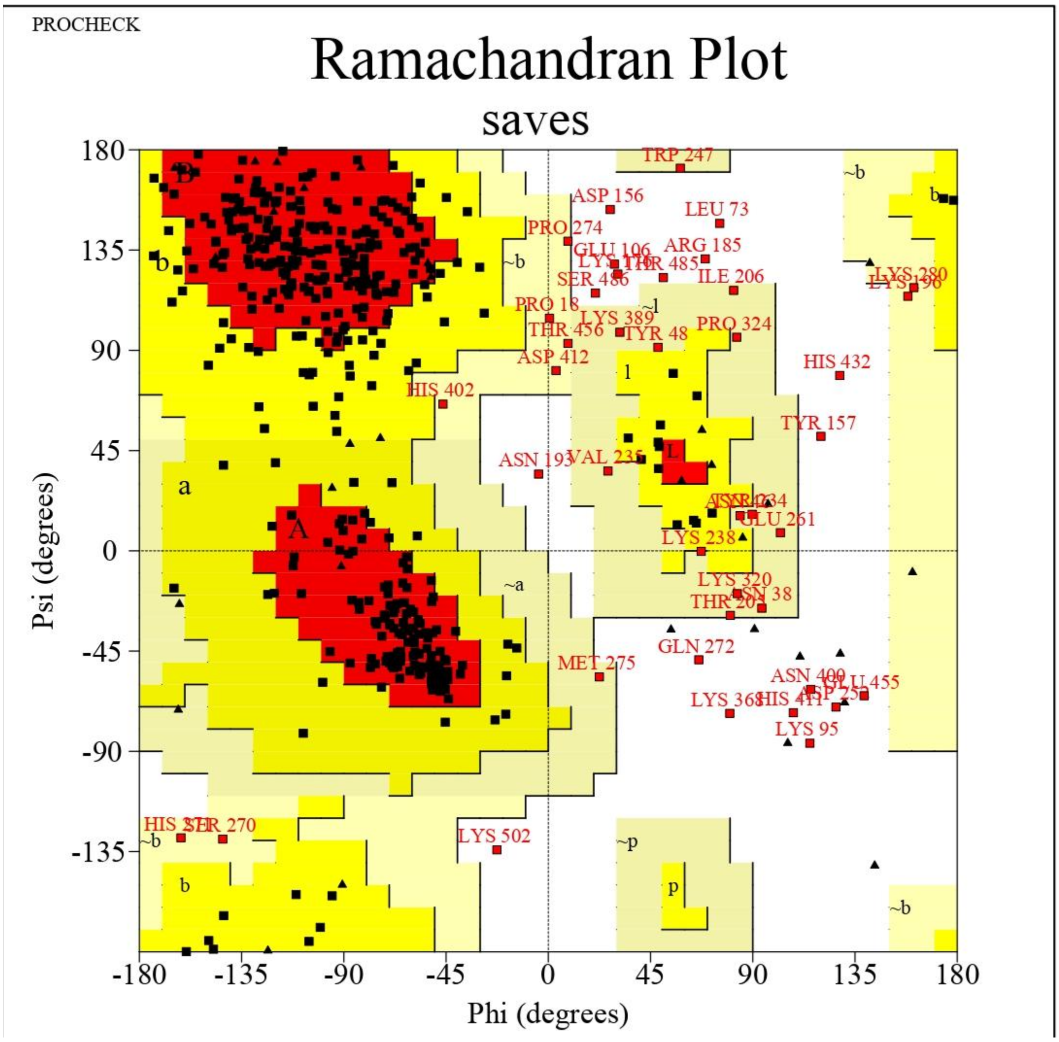

Supplementary Figure 4 (Figure S4) : Evaluation of 3D structure of Hypothetical Protein Q8V4S4 through Ramachandran Plot.

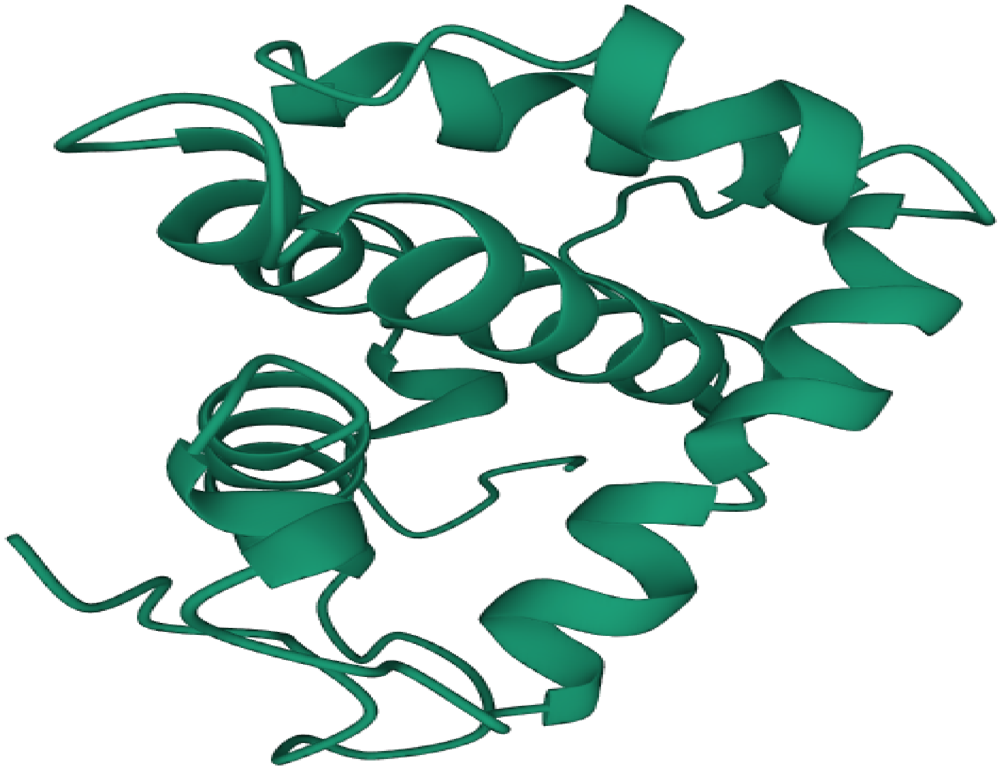

Supplementary Figure 5 (Figure S5) : 3D structure of hypothetical protein Q8V4Q4 predicted from Phyre2.

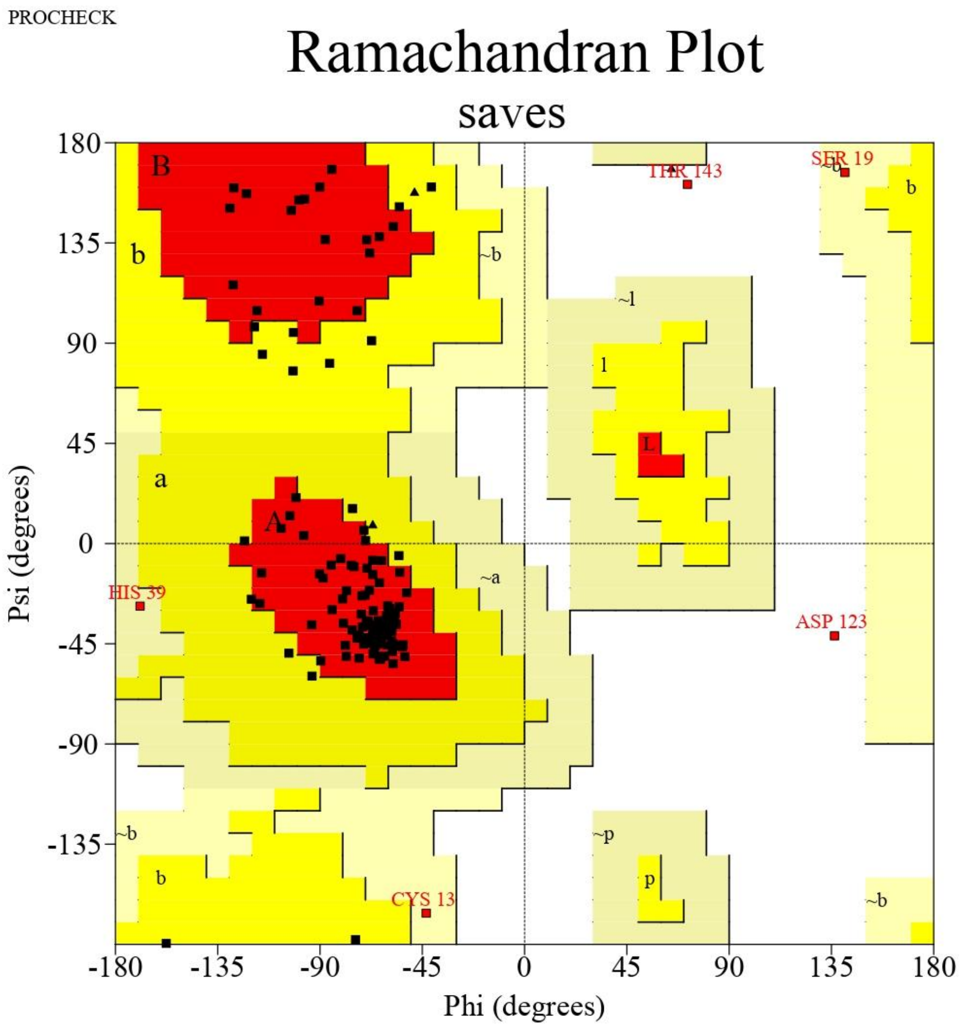

Supplementary Figure 6 (Figure S6) : Evaluation of 3D structure of Hypothetical Protein Q8V4Q4 through Ramachandran Plot.
